# Relationship between gene expression profile class and tumor thickness regression after plaque brachytherapy for choroidal melanoma

**DOI:** 10.1186/s40942-022-00397-x

**Published:** 2022-07-16

**Authors:** Arjun Sharma, John E. Mignano, Jay S. Duker

**Affiliations:** 1 Tufts University School of Medicine, 145 Harrison Ave, Boston, MA 02111 USA; 2 Department of Radiation Oncology, Tufts Medical Center, 800 Washington Street, Boston, MA 02111 USA; 3 New England Eye Center, 800 Washington Street Box 450, 260 Tremont St Biewend Building, 9th-11th Floors, Boston, MA 02116 USA

**Keywords:** Choroid, Melanoma, GEP testing, Plaque brachytherapy

## Abstract

**Background and objective:**

To examine the relationship between gene expression profile class and tumor thickness reduction as measured by ultrasonography in response to plaque brachytherapy using a single-center, retrospective cohort study.

**Methods:**

A total of 15 patients with choroidal melanoma who underwent biopsy for gene expression profiling and were treated with plaque brachytherapy from a single institution from 12/8/14 through 12/19/19 were retrospectively reviewed for clinical characteristics and rate of tumor regression. Ultrasonographic B-scan tumor height was recorded just prior to plaque placement and following plaque removal in the patient’s chart to assess percent reduction in tumor thickness from baseline.

**Results:**

A total of 15 patients met inclusion criteria and were analyzed in this study. Minimum follow-up was 6 months after plaque removal. The percent regression in tumor thickness from baseline as measured by ultrasonography was greater for class 2 tumors than for class 1 tumors at 12-month follow up after treatment, and this difference was statistically significant (P = 0.012). There was no statistical significance in reduction at 3 months (P = 0.46) and 9 months (P = 0.10) after plaque brachytherapy. Although not statistically significant, class 2 tumors appeared to regress more rapidly than class 1 tumors in response to radiation.

**Conclusions:**

In this study, class 2 choroidal melanoma tumors show a more rapid anatomic response to treatment than class 1 tumors at 12 months post plaque brachytherapy.

## Background and objective

Uveal melanoma is the most common primary intraocular tumor in adults with 90% involving the choroid [1]. Plaque brachytherapy remains the prevailing treatment for these tumors because of the ability to administer a highly concentrated dose of radiation to a localized area with minimal impact to surrounding viable tissue [2]. In the absence of histologic or genetic information, clinical parameters have been studied by ocular oncologists in an effort to ascribe subsequent risk of metastases following radiation therapy. Previous studies suggested that faster tumor regression following plaque therapy is associated with a higher risk of mortality due to metastatic spread, offering prognostic value to the clinician early on in the treatment course [3].

Predicting metastatic spread risk has been aided with the advent of gene expression profile (GEP) testing via fine needle aspiration biopsy (FNAB) revealing two distinct genetic tumor types, class I and class 2. GEP testing of tumor cells has been shown to be superior than previous genetic testing looking at only monosomy 3 status to predict metastasis [4]. Past literature demonstrated that class 2 tumors are generally larger, more likely to metastasize, and subsequently result in higher death rates than class 1 tumors [5]. Response to radiation therapy has less consensus within the field with mixed findings relating to tumor thickness reduction after plaque brachytherapy. One large multicenter study found that class 1 uveal melanoma tumors regress more rapidly than class 2 tumors in the 6 months after plaque radiation [2]. Rao et al. had similar findings earlier in the treatment course, with class 1 tumors regressing earlier than class 2 tumors 3 months after I-125 radiotherapy [6]. Other studies, such as ones conducted by Chappell et al. and Augsburger and Correa, found no statistically significant change in mean tumor thickness between class 1 and class 2 tumors 24 months after radiotherapy [7, 8]. This study aims to elucidate the relationship between GEP class and tumor regression after plaque brachytherapy in patients diagnosed with choroidal melanoma.

## Patients/materials and methods

A retrospective, single-institution cohort chart review study was performed on patients treated with plaque brachytherapy and diagnosed with choroidal melanoma (CM) over a 5-year period from 12/3/14 to 12/13/19. Inclusion criteria for the study included: adult patients (> 18 years) seen at the New England Eye Center, pre-operative diagnosis of CM with an initial B-Scan ultrasonography documenting tumor thickness, GEP testing results from Castle Biosciences, and a minimum follow-up of 6 months. Data was entered into electronic health records database. Relevant baseline information such as age, sex, and eye involved was collected with arbitrary patient numbers assigned (Table 1). Clinical data was also recorded, namely AJCC tumor staging, tumor thickness at the start of therapy, and follow-up measurements at 3, 9, and 12-month intervals (± 60 days in each period) via B-scan ultrasound to visualize the posterior eye.

**Table 1:** Patient demographic and clinical characteristics

Patient #	Age	Sex	Eye involved	Initial thickness (mm)	AJCC tumor staging	GEP class
1	60	M	R	5.53	T2aN0M0, stage IIA	2
2	59	M	R	7.4	T2aN0M0, stage IIA	1 A
3	67	M	L	4.4	T3aN0M0, stage IIB	2
4	65	F	L	3.5	T1aN0M0, stage I	1 A
5	62	M	L	4.36	T2aN0M0, stage IIA	1B
6	65	M	R	3.6	T1aN0M0, stage I	1B
7	46	M	R	4	T2aN0M0, stage IIA	1B
8	37	M	R	3.13	T2aN0M0, stage IIA	1A
9	68	M	L	4.11	T1aN0M0, stage I	2
10	77	F	L	4.51	T2aN0M0, stage IIA	1A
11	68	F	R	5.5	T2aN0M0, stage IIA	1A
12	68	F	L	3.73	T1aN0M0, stage I	1A
13	71	F	R	8.67	T3cN0M0, stage IIIA	2
14	73	M	R	3.32	T2aN0M0, stage IIA	2
15	66	M	R	3.96	T2aN0M0, stage IIA	1B

B-scan ultrasound consists of five total views (four transverse/cross-section views and one longitudinal/radial section) with the patient looking either nasally, temporally, up, or down by convention. First, the probe marker is oriented to the corresponding field of interest, then the optic nerve is located, followed by a slow, sweeping motion until the lesion is identified, and finally tumor dimensions are measured.

Fine needle aspiration biopsy was performed prior to plaque placement via the pars plana using a 27 gauge long needle attached to a 10 cc syringe via tubing. In all cases, 25 gauge valved cannulas were used. In most cases a localized vitrectomy was performed prior to biopsy. The creation of a posterior vitreous detachment (PVD), if not already present, was not a part of the surgical technique. Laser was not applied to the biopsy site. If cytologic evaluation was to be performed as well, a second needle was used and, in most cases, the biopsy for cytology was performed via the same retinotomy as was the first biopsy for genetic testing. In all cases, a partial air-fluid exchange was done to isolate the sclerotomy sites from potential tumor cell release into the subconjunctival space. In no cases was cryotherapy applied to the sclerotomy sites. All sclerotomy sites were sutured. Tumors were localized prior to plaque placement using a combination of indirect ophthalmoscopy and transillumination. After final suturing of the plaque, location was reconfirmed using indirect ophthalmoscopy.

## Results

A total of 15 patients were diagnosed with CM and treated with plaque radiation meeting inclusion criteria. The mean age at the time of treatment of the cohort is 63.5 years (range 37–77). 66.67% of patients were men. Ten patients had class 1 tumors and five patients had class 2 tumors. The mean age of the patient at time of treatment for class 1 tumors was 61.3 years old and 67.8 years old for patients with class 2 tumors.

**Fig. 1:**
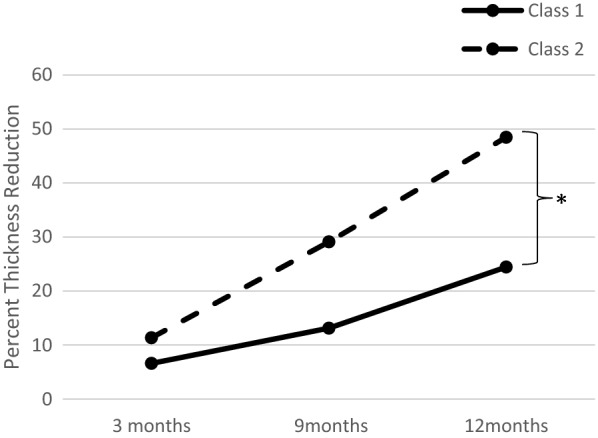
Mean percent tumor thickness reduction after plaque brachytherapy

Mean pretreatment/initial tumor thickness was greater for class 2 patients (5.21 mm) as compared with class 1 patients (4.37 mm). Mean tumor thickness at 12 months post-treatment follow up was smaller for class 2 patients (3.21 mm) as compared with class 1 patients (3.65 mm). Average percent tumor thickness regression for class 1 and class 2 tumors was calculated at 3, 9, and 12-month intervals from B-scan height measurements. Percent reduction was determined by subtracting measured thickness at each interval from the initial tumor thickness and dividing this value by the baseline measurement. A two-tailed, unpaired, equal variance T-test was performed in Microsoft Excel on the mean reduction values, which showed a statistically significant difference (P = 0.012) in height reduction at 12 months post-treatment (Fig. 1).

## Discussion

This study aimed to address the impact of GEP class on tumor regression after plaque radiation. The data presented here illustrates that class 2 CM tumors regress more rapidly than class 1 tumors over a 12-month post-treatment period. The average percent reduction from baseline at each time point measured by B-scan ultrasonography was also greater in class 2 tumors compared with class 1. In addition, the average pre-treatment tumor height was larger in class 2 tumors than class 1.

Mruthyunjaya et al. previously observed that patients with class 2 tumors were significantly older than class 1 patients potentially due to age-related senescence. The patients analyzed in this current study support this relationship [2]. The class 1 patients were an average age of 61.3 years, while patients with class 2 tumors at the time of treatment were 67.8 years of age. Another interesting finding from this study that contradicts previous literature was the difference in early percent reduction after therapy. Two larger studies demonstrated a significantly greater response to plaque therapy in class 1 tumors at 3 and 6-month time points [2, 6]. Two additional reports found a lack of association between GEP class and anatomic tumor response to therapy [7, 8]. Our data shows a different trend, with class 2 tumors regressing faster and at a greater magnitude than class 1 counterparts at a more distant one-year mark. One explanation for our findings relates to differing mitotic rates and metabolic activity between tumor classes. Class 2 tumors are known to carry an increased metastatic risk and aggressive clinical course than class 1 tumors [5]. Since radiation therapy targets actively dividing cells, the more aggressive and active class 2 tumors exhibit greater sensitivity to plaque brachytherapy.

Limitations of this study include the retrospective nature, small sample size, and slight variability between post-treatment evaluation intervals. The overall findings of this study demonstrate a clear association between GEP class and tumor height reduction after plaque brachytherapy. Future investigations should be aimed at recruiting larger patient cohorts with both tumor types to strengthen the association seen here. Clinically, these findings can aid the physician in determining treatment plans and managing patient expectations following observed tumor thickness regression.

## Data Availability

The datasets used and/or analyzed during the current study are available from the corresponding author on reasonable request.
